# Leigh Syndrome: A Tale of Two Genomes

**DOI:** 10.3389/fphys.2021.693734

**Published:** 2021-08-11

**Authors:** Ajibola B. Bakare, Edward J. Lesnefsky, Shilpa Iyer

**Affiliations:** ^1^Department of Biological Sciences, J. William Fulbright College of Arts and Sciences, University of Arkansas, Fayetteville, AR, United States; ^2^Division of Cardiology, Pauley Heart Center, Department of Internal Medicine, School of Medicine, Virginia Commonwealth University, Richmond, VA, United States; ^3^Department of Physiology/Biophysics, School of Medicine, Virginia Commonwealth University, Richmond, VA, United States; ^4^Department of Biochemistry and Molecular Biology, School of Medicine, Virginia Commonwealth University, Richmond, VA, United States

**Keywords:** Leigh syndrome, mitochondria, respiratory chain complex, mitochondrial DNA, mitochondrial genetics

## Abstract

Leigh syndrome is a rare, complex, and incurable early onset (typically infant or early childhood) mitochondrial disorder with both phenotypic and genetic heterogeneity. The heterogeneous nature of this disorder, based in part on the complexity of mitochondrial genetics, and the significant interactions between the nuclear and mitochondrial genomes has made it particularly challenging to research and develop therapies. This review article discusses some of the advances that have been made in the field to date. While the prognosis is poor with no current substantial treatment options, multiple studies are underway to understand the etiology, pathogenesis, and pathophysiology of Leigh syndrome. With advances in available research tools leading to a better understanding of the mitochondria in health and disease, there is hope for novel treatment options in the future.

## Introduction

Leigh syndrome (LS), was first described in 1951 by Denis Archibald Leigh as Subacute Necrotizing Encephalomyelopathy (NSE) and is a complex and incurable early onset neurodegenerative disease (Leigh, 1951). According to the definition used by the Online Mendelian Inheritance in Man Database (OMIM 256000), LS has been defined by these cardinal characteristics: “a neurodegenerative disease with variable symptoms due to mitochondrial dysfunction (caused by hereditary genetic defect) accompanied by bilateral Central Nervous System (CNS) lesions that can be associated with further abnormalities in diagnostic imaging.” Decades of research after the initial description by Denis Leigh have led to the association of LS with defect(s) in one or several of the electron transport chain (ETC) complexes of the mitochondria. Clinical symptoms include neurodevelopmental deterioration, which is often accompanied by brainstem dysfunction such as abnormalities in tone, power, reflexes, ataxia, dysphagia, and seizures (Nesbitt et al., 2012). While the clinical presentations might differ between individuals, LS symptoms largely represent the areas in the brain (brainstem, cerebellum, basal ganglia, oculomotor and cranial nerves) involved in its pathology (Ruhoy and Saneto, 2014).

Like most other mitochondrial diseases, LS is clinically and genetically heterogeneous, resulting in a diverse phenotypic spectrum. Due in part to mutant load, LS can present as NARP (Neuropathy, ataxia, and retinitis pigmentosa) at low mutant loads between 50–60%; while phenotypically present as LS at higher mutant loads >90%. The heterogeneous nature of LS can also be attributed in part to the complex nature of the mitochondrial ETC, which is composed of subunits that are encoded by both nuclear (nDNA) and mitochondrial DNA (mtDNA) (Nesbitt et al., 2012; Zinovkin et al., 2016; Bailey and Doherty, 2017; Manickam et al., 2017) with mutations in either genomes coding for different ETC subunits resulting in LS. Furthermore, since the mitochondria do not follow the Mendelian inheritance pattern, healthy and diseased mtDNA could co-exist within a cell (Taylor et al., 2001b). This phenomenon, termed heteroplasmy, also contributes to the complexity and diverse phenotypic spectrum characteristic of LS. In some cases, it is believed that a certain heteroplasmic threshold must be attained for the expression of the diseased phenotype.

Here, we review the current knowledge of LS, describing how mutations in various ETC subunits encoded by either nDNA or mtDNA contribute to disease pathogenesis. Emphasis will be placed on the most common mutations that have been reported to result in deficiencies of each of the complexes that comprise the OXPHOS system. This review will also discuss some of the disease models that are currently being used to study LS, the challenges associated with these models, and the potential for induced pluripotent stem cell (iPSC) technologies contributing to novel models for understanding the pathophysiology of LS. Finally, current and potential treatment options for LS will also be discussed.

## Background of Mitochondrial Biology and Leigh Syndrome

### Basics of Mitochondria

Mitochondria are key organelles that are critical to normal cell and organ function and serve an essential role in maintaining metabolic homeostasis through the production of energy in the form of Adenosine Triphosphate (ATP) (Graeber and Muller, 1998; Shaughnessy et al., 2014; Zinovkin et al., 2016). Mitochondria also serve as the hub for various cellular activities such as lipid metabolism, the citric acid cycle, and oxidative phosphorylation (OXPHOS) (Graeber and Muller, 1998). Mitochondria produce ATP via the OXPHOS pathway; a process that takes place at the inner mitochondrial membrane and involves the channeling of electrons through four ETC complexes. The electron transport results in subsequent translocation of protons from the matrix into the intermembrane space; with the combination of the proton gradient and the inward-negative mitochondrial membrane potential driving the molecular motor, ATP synthase (complex V), to produce ATP. An impairment in the ETC or its assembly complexes (Supplementary Table 1) results in metabolic disorders like LS. The ETC, as the name suggests is a vast complex comprising of approximately 90 different subunits which make up the four enzyme complexes and complex V of OXPHOS, with the mtDNA encoding 13 subunits and the rest encoded by nuclear DNA (nDNA) (Larsson and Clayton, 1995; Koopman et al., 2013). Together, the nDNA and mtDNA coordinate the synthesis of subunits that come together to form the individual complexes that comprise the ETC, subsequently allowing the mitochondria to function as the core energy producer for cellular needs.

**FIGURE 1:**
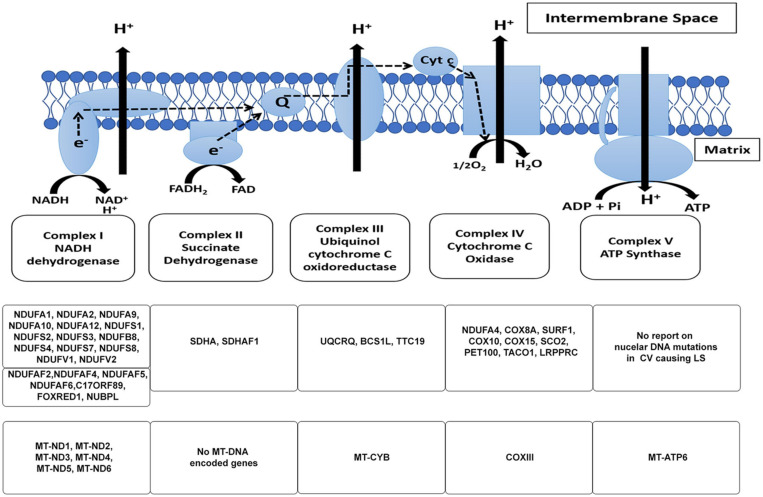
The mitochondrial electron transport chain. The subunits of the respiratory chain are encoded by nDNA and mtDNA. Mitochondria produce ATP via the OXPHOS pathway; a process that takes place at the inner mitochondrial membrane involves the channeling of electrons through four-electron transport chain (ETC) complexes (Complex I–IV). The electron transport results in subsequent translocation of protons from the matrix into the intermembrane space, this proton gradient in combination with the inward-negative mitochondrial membrane potential drives the molecular motor, ATP synthase (complex V), to produce ATP. An impairment in the ETC or its assembly complexes results in metabolic malfunction in cells and tissues. The ETC is a vast complex comprising ∼90 different subunits which make up the five enzyme complexes of OXPHOS. Of these subunits, mtDNA encodes 13 subunits while the nuclear DNA (nDNA) encodes ∼77 subunits. Together, the nDNA and mtDNA coordinate the synthesis of subunits that come together to form the individual complexes that compose the ETC, subsequently allowing the mitochondria to function as the core energy producer for cellular needs. Specific genes encoded by nDNA and mtDNA in the context of LS are listed.

The human mitochondrial DNA is a 16.6 kb pair, double-stranded circular genome containing 37 genes. These genes encode for: 13 subunits (Figure 1) of the ETC and 24 genes involved in mtDNA translation [2 ribosomal RNAs (rRNAs) and 22 transfer RNAs (tRNAs)] (Larsson and Clayton, 1995; Graeber and Muller, 1998; Wallace, 1999; Taylor et al., 2001b; Dimauro and Schon, 2003; Koopman et al., 2013). The remainder of the mitochondrial ETC and associated assembly factor proteins, replication, transcription, translation, and regulatory proteins are encoded by the nDNA (see Table 1; Wallace, 1999; Iyer et al., 2009a). It is widely accepted that all mitochondrial organelles originated from an early endosymbiont event involving an α-protobacterium and a nucleus containing host cell (Gray et al., 1999; Dimauro and Schon, 2003; van Esveld and Huynen, 2018). During evolution, this early endosymbiotic relationship became so intertwined that these organisms became very dependent on one another thus requiring the streamlining of the genome of the protobacterium to create a more efficient organelle (Wallace, 1999; van Esveld and Huynen, 2018). The modern human mitochondria is a product of this evolutionary event; with the loss of some of the early protobacterium genes and insertion of other genes into the host’s genome (van Esveld and Huynen, 2018). As a result, the ETC is under the control of two genomes. Therefore, mitochondrial genetics is governed by inheritance patterns, which are slightly different from Mendelian genetics.

**TABLE 1:** The electron transport chain complexes (ETC) with subunits encoded by mitochondrial (mtDNA) and nuclear DNA (nDNA).

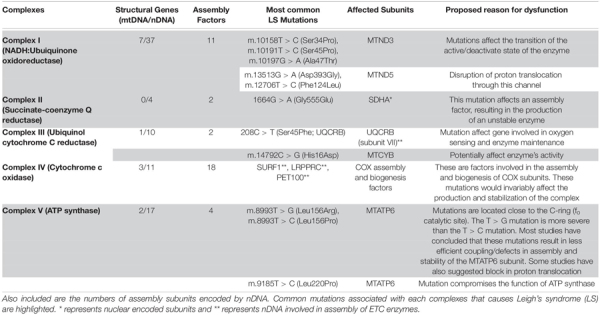

During the electron transfer to molecular oxygen, reactive oxygen species (ROS) are generated by leakage of electrons in complex I and III causing oxidative stress to cells (Mitchell, 1961; Murphy, 2009; Nicholls and Ferguson, 2013). ETC defects occurring from mtDNA or nDNA mutations compromise membrane potential and ATP synthesis, and interruption of this pathway renders cells and tissues vulnerable under disease and oxidative stress conditions (Iyer et al., 2012; Jain et al., 2016). Consequently, an error in either nDNA or mtDNA encoding for proteins that make up any of the subunits could result in disorder(s) of the ETC and the mitochondria. Advances in next-generation and whole-exome sequencing continue to reveal novel nDNA mutations involved in LS; with subsequent inheritance in an autosomal recessive or X-linked inheritance (vs. maternal) pattern. Owing to its ubiquitous role, the mitochondria are present in all human cells (except red blood cells), and their distribution and copy number vary from cell to cell. ETC defects impairing OXPHOS result in major pathological problems for energy-demanding cells and tissues such as those of the heart, brain, and muscles (Ruhoy and Saneto, 2014; Maglioni et al., 2020). Not surprisingly, many of the symptoms associated with LS and similar metabolic disorders affect the neuromuscular or cardiovascular systems (Leigh, 1951; Ruhoy and Saneto, 2014; Veerapandiyan et al., 2016). While it is widely accepted that both the nDNA and mtDNA contribute to the normal function of the mitochondrial ETC, the mechanism underlying cross-talk and communication between these genomes in the diseased state remains under investigation. While this review focuses largely on mitochondrial dysfunction resulting from pathogenic mutations affecting ETC enzymes, mutations resulting in defective mitochondrial translation machinery (Sweeney et al., 1994; Sue et al., 1999; Antonicka et al., 2003; Tucker et al., 2011; Cox et al., 2012; Ghezzi et al., 2012; Kopajtich et al., 2014; Suzuki and Suzuki, 2014; Tischner et al., 2015), non-ETC enzymes such as pyruvate dehydrogenase (Matthews et al., 1993; Finsterer, 2008; Patel et al., 2012), or cofactors (Gerards et al., 2013; Banka et al., 2014; Smith et al., 2018) can affect OXPHOS and also result in mitochondrial dysfunction.

### Mitochondrial Genetics and Heteroplasmy

Unlike Mendelian genetics, mtDNA inheritance follows some unique inheritance patterns. Maternal inheritance, heteroplasmy, and mitotic segregation are some of the traits that separate mitochondrial genetics from those of Mendelian genetics (Wallace, 1999; Dimauro and Schon, 2003). During normal human development, only mtDNA from the mother is inherited by the offspring (Wallace, 1999). Although there have been rare reports of mtDNA inheritance from the father, this is usually not the case; as mtDNA from the sperm are usually degraded through an unknown mechanism during fertilization (Sutovsky et al., 1999; Schwartz and Vissing, 2002). Therefore, a mother with mutant mtDNA will always pass this to her children and the daughter of this mother will pass this along to her offspring as well. This inheritance pattern termed maternal inheritance is largely involved in cases of maternally inherited mitochondrial disorders such as MILS (Maternally Inherited Leigh Syndrome).

Within a cell, there are hundreds of mitochondria, with each mitochondrion having multiple copies of mtDNA, and a cell comprising of thousands of copies of mtDNA (Wallace, 1999; Dimauro and Schon, 2003; Tachibana et al., 2009). In the first steps of human development, the mitochondria inherited by an individual are derived exclusively from the oocyte during fertilization. In healthy offspring, the inherited mitochondria will contain copies of the same wild-type mtDNA, referred to as homoplasmy. However, owing to its proximity to sites of reactive oxygen species (ROS) production in the mitochondrial matrix, and an error-prone polymerase, mtDNA has a very high mutation rate (Richter et al., 1988; Wallace, 1999; Shaughnessy et al., 2014). Initially, when a mutation occurs, cells will contain a mixture of wild-type and mutant mtDNA, a phenomenon referred to as heteroplasmy. Heteroplasmy can occur at both cellular (and tissue) and organelle levels, just as a cell can harbor mitochondria with wild-type and mutant mtDNA, a process that can occur within a mitochondrion as well. Since mtDNA division does not coincide with nDNA division (Larsson and Clayton, 1995); a division of heteroplasmic cells that occurs by a process known as replicative/mitotic segregation, could invariably shift the mtDNA genotype of the daughter cells to those of the mutant or wild-type mtDNA, over many generations (Wallace, 1999). Therefore, heteroplasmy in combination with replicative segregation can shift the phenotype of a healthy cell to a diseased state. For a mutation to be pathogenic, however, the percentage of mutant mtDNA must exceed a threshold such that oxidative metabolism is affected. If the percentage of mutant mtDNA continues to increase, this will consequently result in a gradual and continuous decrease in energy production until a bioenergetic threshold is reached (Wallace, 1999). Above this threshold, cellular needs cannot be met, invariably, resulting in disease phenotype that is usually characteristic of metabolic disorders such as LS. This phenomenon referred to as the threshold effect is responsible for the clinical signs and heterogeneity in disease penetrance observed in mitochondrial disorders. Tissues that are metabolically active and highly dependent on oxidative phosphorylation – such as the brain, heart, skeletal muscles, retina – tend to have a lower threshold; and are therefore more vulnerable and less tolerant to the pathogenic effects of mtDNA mutations (Dimauro and Schon, 2003).

The variation in copy number and stochastic nature of mitochondrial genetics results in a variable inheritance and expression pattern of heteroplasmic mutations; and largely contributes to the variability and spectrum of disease phenotype associated with LS. The genotype-phenotype relationship in LS patients is difficult to ascertain because different heteroplasmic loads exhibit different phenotypes. For example, m.8993T > G (m.8993T > C) mutation; a mutation that affects mt-DNA encoded ATP synthase 6 (ATPase 6) gene, when present in low abundance only results in NARP (neurogenic muscle weakness, ataxia, and retinitis pigmentosa), while a high abundance of the same mutation results in MILS (Maternally Inherited Leigh’s Syndrome) with rapid lethalities (Iyer et al., 2012). Furthermore, there have been reports of mutations in OXPHOS genes encoded by nDNA presenting with similar phenotypes to those of mtDNA mutations; and cases of different genetic mutations resulting in the same diseased phenotype.

### What Is Leigh Syndrome?

Mitochondrial (mt) disorders represent a large group of severe genetic disorders mainly impacting organ systems with high energy requirements (McFarland et al., 2010; Maglioni et al., 2020). These disorders are clinically complex, often fatal, and occur at an estimated ratio of 1 in 5,000 to 10,000 live births (Skladal et al., 2003; Schaefer et al., 2004). Leigh syndrome (LS) is a classic example of mitochondrial disorder resulting from pathogenic mutations that disrupt OXPHOS capacities. Although first described in 1951, it was not until 1968 when Hommes and colleagues described a case of a one-year-old patient that LS first became associated with defects in mitochondrial energy metabolism (Hommes et al., 1968). By 1983, multiple reports resulted in the first description of LS as a mitochondrial disease (Willems et al., 1977; van Erven et al., 1987; Hammans et al., 1991). During the early years after its initial description, its resemblance to Wernicke’s encephalopathy (WE) led many to believe that LS resulted from an error in thiamine metabolism (Worsley et al., 1965; Baertling et al., 2014; Leigh et al., 2015). Many years after its first description, it was noted that some patients with LS presented with deficiencies in either the pyruvate decarboxylase (Evans, 1981) or pyruvate dehydrogenase complex (PDHc) (Devivo et al., 1979). These variations in disease phenotype allude to the heterogeneous nature of LS.

During its initial description, the characterization of pathological hallmarks of LS was diagnosed postmortem by histopathological observations. However, advances in sequencing, biochemical, and imaging technologies have resulted in better antemortem diagnoses. Furthermore, in the last 15 years, these technologies have resulted in the identification of novel pathogenic mutations in LS patients, with more than 75 genes identified as monogenic causes of LS (Lake et al., 2016). Based on genetic analysis, LS can be inherited in any of these patterns: maternal inheritance, autosomal recessive inheritance, very rare X-linked or autosomal dominant inheritance, sporadic *de novo* mutation and through complex heterozygosity (Ruhoy and Saneto, 2014; Leigh et al., 2015; Lake et al., 2016).

### Clinical Presentation and Diagnostic Approaches for Leigh Syndrome

**FIGURE 2:**
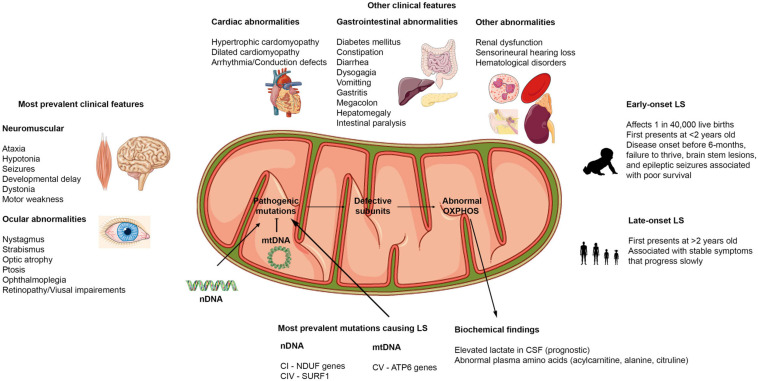
Clinical features of Leigh syndrome. Figure showing the various clinical features associated with LS. The most prevalent clinical features affect the brain, muscles, and eyes. Other clinical findings include dysfunctions in cardiovascular, gastrointestinal, renal, auditory, and hematological systems. LS can present as early onset or late-onset with abnormalities in at least 3 of the organ systems highlighted. LS results from pathogenic mutations in either the nDNA or mtDNA that causes abnormalities in the OXPHOS capacities of the mitochondria. Hence, biochemical findings reflect these defects.

As highlighted above, LS is highly heterogeneous, owing to many of the factors discussed previously. There is a continuous effort in both the clinical and scientific research fields to reconcile the relationship between the genetic and phenotypic presentations reported in patients with LS (Figure 2). There are various case reports describing some of the clinical symptoms of LS; however, because of how rare this disorder is, the small sample sizes in these reports restricts analytical studies (Uittenbogaard et al., 2018; Wei et al., 2018; Yu et al., 2018; Edwards et al., 2020). This makes its challenging to fully identify the most predominant symptoms in patients with LS. In recent years, however, efforts have been made to compile clinical information from various centers across Europe, Asia, the United States, and Australia (Sofou et al., 2014, 2018; Ganetzky et al., 2019; Hayhurst et al., 2019; Hong et al., 2020; Ogawa et al., 2020; Stendel et al., 2020). These have allowed for metadata analysis and systemic retrospective studies to find unifying symptoms for LS.

Although LS shows clinical heterogeneity, the most prevalent symptoms that have been reported correlate with the involvement of brain regions such as the basal ganglia, brainstem, thalamus, and cerebellum (Yu et al., 2018; Chang et al., 2020). These regions of the brain control body movement, balance, and basic life functions like breathing, swallowing, and blood circulation (Chang et al., 2020). In most patients with LS, pathological lesions in one or more of these areas of the brain have been found in MRI (Magnetic Resonance Imaging) reports, contributing to observed clinical manifestations of LS (Baertling et al., 2014). It is worth noting that, while pathological lesions in basal ganglia and brainstem are considered the hallmarks of LS, there have been reports of cases where basal ganglia (Sonam et al., 2014) or brainstem lesions (Hayhurst et al., 2019) were not detected in patients with LS (Stendel et al., 2020). However, other neuropathological signs such as delayed myelination, cerebral atrophy, and cerebellar lesions were reported in these cases (Sonam et al., 2014; Chourasia et al., 2017; Stendel et al., 2020). These findings suggest that LS can present primarily with pathological lesions in other regions of the brain and can predate other neuroimaging features that are considered hallmarks of LS. While these findings demonstrate the need to consider expanding the diagnostic criteria for LS on neuroimaging findings, they also highlight one of the limitations of neuroimaging as a diagnostic tool for LS. Neuroimaging findings need to be combined with other diagnostic tools such as biochemical, histopathological, and sequencing information to properly diagnose LS.

To date, the most common clinical features associated with LS are (see Figure 2 for more details): ataxia, hypotonia, developmental delay, seizures, poor feeding/feeding difficulties associated with dysphagia, failure to thrive, persistent vomiting, elevated serum or cerebrospinal fluid lactate levels, and abnormal ocular disturbances (Gerards et al., 2016; Lee S. et al., 2019; Chang et al., 2020; Hong et al., 2020; Ogawa et al., 2020; Stendel et al., 2020). Aside from the neuromuscular and ocular abnormalities, abnormalities in other organ systems have also been reported in some cases (Figure 2). During the course of the disease, some patients present with gastrointestinal and cardiac problems (Gerards et al., 2016; Sofou et al., 2018). In addition, respiratory distress has been reported to be a common clinical feature in patients with early onset LS – before the age of two (Yu et al., 2018). Recent reports have suggested that certain clinical features are more common in early onset cases than in late-onset cases of LS. In a study performed in Korea to distinguish clinical features of early and late-onset LS, it was found that developmental delay was significantly higher in the early onset (before 2 years old) compared to the late-onset (after 2 years old) groups. Motor weakness and ataxia were predominant in the late-onset group relative to the early onset LS patients (Yu et al., 2018; Hong et al., 2020). Further, the onset of disease could also dictate the type of seizures patients present with (focal or generalized seizures) (Wei et al., 2018). The presence of pathological signs at birth and history of epileptic seizures have been strongly associated with poor prognosis (Sofou et al., 2014; Hong et al., 2020). Because LS induces alteration in mitochondrial activities, many biochemical findings focus on lactic acid levels in serum and cerebrospinal fluid (CSF). However, conflicting reports on lactate levels have been reported, with some reports of high serum and CSF lactate in LS patients (Sofou et al., 2014; Uittenbogaard et al., 2018; Edwards et al., 2020), while others have reported normal serum and CSF lactate levels in some other patients (Gerards et al., 2016). New evidence suggests that elevated lactate levels could also be associated with disease onset. Elevated lactate in CSF was more common and significantly correlated to a more severe disease course and associated with patients with early onset (before 6-months) (Sofou et al., 2014; Yu et al., 2018). Therefore, instead of using lactate levels as a diagnosis for LS as previously suggested (Rahman et al., 1996), it could serve as a prognosis to determine the severity of the disease in patients.

Histopathological findings with muscle biopsies have been another source of controversy in defining clinical features of LS. While positive muscle biopsy findings such as COX, SDH deficiency (Sonam et al., 2014), ragged red fibers (RRF), and atrophy of muscle fibers (Sofou et al., 2014) have been used as a diagnosis for LS, non-specific myopathic changes and negative findings have also been reported in patients with LS (Wei et al., 2018). Furthermore, findings such as RRF are associated with another mitochondrial disorder: myoclonus epilepsy with ragged red fibers (MERRF). Finally, biochemical findings highlighting deficiencies in respiratory chain complexes, while important in LS diagnosis have also been inconsistent (Ganetzky et al., 2019; Ogawa et al., 2020). As stated earlier, these observations highlight the need to combine findings from various diagnostic tools including genetic findings for proper diagnosis of LS.

Although the clinical presentations of LS are heterogeneous, the collaborative efforts resulting in the collation of clinical data from various sources and the resulting extensive meta-data analysis have provided us valuable information on the most common clinical features associated with LS (Figure 2). Most importantly, we are beginning to understand the mutations most involved in specific clinical features and the association between these features and patient survival. For example, in one study, patients with NDUF mutations had a prevalence of early cerebral cortex abnormalities, high occurrence of cardiac and ocular manifestations relative to other LS patients. In the same study, patients with the mtDNA mutation m.8993T > G had more severe clinical and radiological manifestations and poorer disease outcomes compared to those with the m.8993T > C variant (Sofou et al., 2018). Another study showed that SURF1 deficiency has a more favorable survival outcome (Wedatilake et al., 2013; Sofou et al., 2014). These suggest that there are certain clinical manifestations and phenotypes that might be shared among LS patients with similar mutations and that some mutations have milder disease progression. A better understanding of the genotype and phenotype relationship involved in LS could help with better diagnosis and early treatment of the disease, subsequently resulting in prolonging the lives of patients with LS. As highlighted above, there have been discrepancies in some of the neuroimaging, histopathological, and biochemical findings reported for patients. Some of these differences could potentially result from variability associated with the stage of the disease. As mentioned, some clinical presentations are more prevalent in early onset compared to late-onset LS patients and vice versa. Therefore, the discrepancies recorded could be associated with the progression of the disease. Future longitudinal studies exploring changes in clinical presentation in various cohorts of LS patients could prove invaluable in solving this problem. Since LS is rare, and most patients with the disorder have to be under close supervision, it is difficult to have a controlled study with a very large population size. Furthermore, because there are no standardized ways of diagnosing the disorder, the same tests are not performed for every patient; and thus further confounds our ability to make connections between the clinical presentations and genetic mutations. However, the current approach by the multi centers will continue to add to our understanding of the disorder. It will be beneficial to understand the effects that different mitochondrial haplotypes have on clinical presentations as well. For instance, it has been reported that environmental factors and polymorphism in mtDNA haplogroups J1c and J2c are associated with increased penetrance of LHON (Leber’s hereditary optic neuropathy) (Carelli et al., 2006).

## Factors Influencing Leigh Syndrome

As previously described, LS results from mutations that cause perturbation to the ETC. Once the ETC becomes overburdened and dysfunction in oxidative processes in the mitochondria persist, various pathogenic processes are initiated (Figure 2). As a hub for oxygen and electron-rich compounds, perturbation to the flow of electrons and protons could invariably result in reactive oxygen species and superoxide formation. Consequently, leading to the initiation of stress and inflammatory responses, chronic forms of these responses could result in cell death, culminating in the development of the symptoms associated with LS. In this section, we introduce the ETC and how defects in any of these complexes contribute to LS pathology. We focus largely on the ETC defects resulting from mtDNA mutations that have been reported to be involved in most cases of LS to date. We end this section with a summary of other deficiencies caused by nuclear-encoded genes, not related to ETC enzymes that have also been reported in patients with LS.

### Complex I

Complex I also known as NADH:Ubiquinone oxidoreductase (Figure 1), is the first component and largest complex of the mitochondrial ETC, comprising more than 45 subunits (Sarzi et al., 2007; Nesbitt et al., 2012). The mtDNA encodes only 7 (MTND1-MTND6, MTND4L) of these subunits, while nDNA encodes the remaining structural and assembly factor subunits (Sarzi et al., 2007; Table 1). This enzyme oxidizes NADH (Nicotinamide Adenine Dinucleotide)-produced through glycolysis, the Krebs cycle, and β-oxidation – to reduce ubiquinone (Q); the energy from this redox reaction is coupled to proton translocation across the mitochondrial inner membrane (Sarzi et al., 2007; Nesbitt et al., 2012; Babot and Galkin, 2013; Babot et al., 2014). Owing to its size and location in the OXPHOS pathway, a complex I defect could result in severe respiratory chain dysfunction and has been reported to account for most cases of LS (Kirby et al., 2003). Complex I deficiency can present at any age, with symptoms ranging from isolated myopathy or liver disease to multisystemic disorders (Werner et al., 2009).

To date, approximately 14 nDNA genes encoding for both structural and biogenesis/assembly factor subunits of complex I (Supplementary Table 1) have been implicated in the etiology of LS (Berger et al., 2008; Koopman et al., 2013). Only three mtDNA genes (*MTND2*, *MTND3*, *MTND5*), however, have been reported to be largely involved in LS, with *MTND3* and *MTND5* mutations being the most prevalent of all three mtDNA mutations reported thus far. While these mutations are the most common, there have also been isolated reports of patients with *MTND1*, *MTND4*, and *MTND6* mutations resulting in LS or Leigh-like syndrome, albeit, in very rare cases (McFarland et al., 2004). Some of the most recurrent structural subunit mutations which have been reported to contribute to LS include m.10158T > C (McFarland et al., 2004), m.10191T > C (Taylor et al., 2001a), and m.10197G > A (Sarzi et al., 2007) in the *MTND3* gene; m.12706T > C (Taylor et al., 2002), m.13513G > A (Chol et al., 2003), and m.13514A > G (Corona et al., 2001) in the *MTND5* gene; and m.14459G > A (Kirby et al., 2000), and m.14487T > C (Ugalde et al., 2003) in the *MTND6* gene (Supplementary Table 2).

Both m.10158T > C, and m.10191T > C mutations result in amino acid substitutions of a polar serine residue for a hydrophobic proline residue at codons 34 and 45 of the *MTND3* genes respectively. The third mutation, m.10197G > A results in a substitution of a hydrophobic alanine residue for a polar threonine residue at codon 47 of the *MTND3* gene (McFarland et al., 2004; Sarzi et al., 2007). Structural analysis of the MTND3 subunit suggests that all of these mutations reside in the transmembrane domain of the *MTND3* gene which projects into the mitochondrial matrix (Sarzi et al., 2007). The location of these mutations suggests that they either act to cause dysregulation in complex I assembly or reduce the enzymatic activity of the enzyme. Studies strongly suggest that, although m.10158T > C, and m.10191T > C mutations moderately reduced complex I assembly, the decrease is almost negligible when compared to the drastic reduction in enzyme activity of the complex in these mutants (McFarland et al., 2004), thus suggesting that *MTND3* mutations could result in LS through a decrease in the enzymatic activity of complex I.

Studies have reported LS cases resulting from mutations in m.13513G > A and m.12706T > C of the *MTND5* genes associated with complex I (Kirby et al., 2003). Although it has been reported that disease phenotype only presents in tissues such as muscle and brain at high mutant loads (typically above 90%) for most pathogenic mutations, the *MTND5* gene mutation can result in diseased phenotype at low mutant loads even below 50% (Kirby et al., 2003). This could potentially be a result of very low heteroplasmy; alternatively, the origin (fibroblast) of the cells used in this analysis could potentially explain this observation. Immunoprecipitation studies allude to MTND5 subunit as being located peripherally in complex I, and been suggested to be the last subunit to be assembled into the complex. Further, the MTND5 synthesis is believed to be a rate-limiting step for the activity of complex I (Liolitsa et al., 2003). Therefore, the MTND5 subunit is a key regulator of complex I assembly and stability, consequently is a key modulator of cellular respiration. Taken together, this could explain why a mutation in MTND5 subunits at lower mutant load could result in severe cases of LS and other complex I related disorders such as MELAS (mitochondrial encephalomyopathy with lactic acidosis and stroke-like episodes) or LHON (Leber’s hereditary optic neuropathy).

Given the importance of Complex I in the OxPhos pathway, any mutation that affects its function could gravely perturb the ETC, resulting in decreased ATP synthesis and increased production of ROS and other reactive species. These effects result in a vicious cycle that results in mitochondrial degradation and subsequent cell and tissue death. Studies have suggested that complex I activity has to be reduced by more than 70% before oxygen consumption or ATP production is perturbed (Sarzi et al., 2007), indicating that a high percentage of mutant mtDNA must be present for biochemical or clinical manifestation of disease. However, as mentioned previously, the location of the mutation could determine what percentage of mutant load would result in a diseased phenotype. For example, *MTND3* mutation requires a higher mutant load (greater than 80%) to result in LS phenotype, while *MTND5* mutation could result in LS phenotype at mutant load as low as 50%. Aside from mutation threshold, factors such as additional genetic factors, cell cycle (mitotic cells vs. non-mitotic cells), can influence the expression of complex I deficiency (Sarzi et al., 2007). It is worth noting that recent findings from interventional studies have shown improvement in bioenergetics functions without rescuing ETC defects. Intervention strategies such as those targeting NAD + metabolism, or mTOR inhibition have alleviated mitochondrial disease in some models of LS and related mitochondrial disorders (Johnson et al., 2013; Lee C. F. et al., 2019; Cheema et al., 2021). These findings again allude to the complexity associated with studying mitochondrial disorders.

### Complex II

Complex II, also known as succinate-coenzyme Q reductase, is the smallest of the ETC complexes with all four subunits (Succinate dehydrogenase subunits A to D) being nuclear-encoded (Table 1; Ruhoy and Saneto, 2014). Located in the inner membrane of the mitochondria, this complex participates in both the citric acid cycle and ETC. The largest catalytic subunit, SDHA oxidizes succinate and couples this to the reduction of its flavine cofactor, FAD; while the other catalytic subunit, SDHB shuttles electrons to ubiquinone in a concerted manner (Renkema et al., 2015). Mutations in complex II account for a very small portion of OxPhos disorders, as dysfunction in this complex, is very rare (Pagnamenta et al., 2006; Ohlenbusch et al., 2012; Jain-Ghai et al., 2013; Renkema et al., 2015). More than 10 different autosomal-recessive pathogenic mutations in SDHA have been reported to cause LS, Leigh’s-like, and other related mitochondrial disorders, while mutations in SDHAF1 (a complex II assembly factor), SDHB, and SDHD have also been reported to cause LS or LS-like symptoms (Supplementary Table 1). However, there is still no report of SDHC involvement in the etiology of LS (Renkema et al., 2015). Although clinical phenotype and MRI findings have been used to describe LS or LS-like symptoms in patients with complex II defects, some of the patients presented with other symptoms that were not characteristics of LS (Jain-Ghai et al., 2013). For instance, a case of mild LS was reported to be caused by homozygous G555E mutation in the SDHA subunit of complex II; whereas, this same mutation has been reported to be responsible for cases of lethal-infantile presentations of mitochondrial complex II defects (Pagnamenta et al., 2006). While it is typical to observe phenotypic heterogeneity in heteroplasmic mutations of mtDNA, this is atypical of a nuclear mutation. This observation alludes to the heterogeneous nature of this disease that contributes to its complexity, thus making it difficult to completely understand its etiology.

### Complex III

Mitochondrial complex III, also known as ubiquinol cytochrome c reductase (Figure 1), is located within the mitochondrial inner membrane where it catalyzes the transfer of electrons from succinate and NADH dehydrogenases to cytochrome *c* (de Lonlay et al., 2001; Barel et al., 2008). Further, energy from this electron transfer is used in the translocation of a proton across the inner membrane, contributing to the proton gradient required for OXPHOS. Complex III consists of 11 structural subunits (Table 1) with only one of these subunits, cytochrome *b* (*MT*-*CYB*), encoded by the mtDNA. Isolated mitochondrial complex III deficiencies are rare and present with heterogeneous clinical symptoms characteristic of LS. In a clinical report of a 15-month-old female LS patient of European descent, two homoplasmic mutations involving m.14792C > G and m.14459G > A was reported that resulted in p.His16Asp change in MT-CYB and p.Ala72Val substitution in the *ND6* subunit, respectively (Ronchi et al., 2011). Interestingly, the mother of this patient (normal) was homoplasmic for the m.14792C > G mutation (*MT*-*CYB*) but heteroplasmic for the m.14459G > A mutation. This suggests that the *ND6* mutation and not the MT-CYB mutation is involved in the LS pathology in this patient. While mutations in nuclear-encoded proteins of complex III have been reported to be involved in LS pathology (Supplementary Table 1), most pathological cases of *MT-CYB* mutations (Supplementary Table 2) result in skeletal muscle weakness, exercise intolerance, and in some cases sporadic myopathy-rhabdomyolysis associated with ragged-red fibers (Barel et al., 2008).

While various pathogenic mutations in the gene encoding cytochrome *b* (*MT-CYB*) have been reported, mutations in nDNA have only been reported in three genes to date: the *BCS1L*, *TTC19*, and the *UQCRB* gene (ubiquinol-cytochrome *c* reductase binding protein) (Barel et al., 2008). Both *BCS1L* and *TTC19* genes encode assembly factor proteins of complex III, with the *BCS1L* gene encoding for a complex III assembly factor (de Lonlay et al., 2001), while *TTC19* encoding for tetratricopeptide 19, a protein embedded in the inner mitochondrial membrane (Atwal, 2014). The *UQCRB* gene encodes subunit VI (chromosome 8q22) of complex III, while another study described a large consanguineous inbred Israeli Bedouib kindred with a mutation in the *UQCRQ* gene, which resulted in a c.208C > T mutation in exon2 of the subunit (Barel et al., 2008). This mutation resulted in a substitution of serine for phenylalanine at position 45 (p.Ser45Phe) in the encoded protein, UQCRQ, complex III subunit VII and resulted in LS-like symptoms. In addition to decreased activity of complex III, the activity of complex I was also decreased in these patients, a common observation because of the structural interdependence of complex I and complex III. While *TTC19* mutations are rare, *BCS1L* constitutes most cases of LS associated with nDNA mutations (Atwal, 2014). As has been alluded to previously, both isolated complex III and combined complex I and III deficiencies can contribute to LS pathology. Thus, future work needs to focus on understanding supercomplex assembly and dysregulation in this assembly as it relates to LS.

### Complex IV

Also referred to as cytochrome c oxidase (COX), complex IV is the terminal enzyme of the mitochondrial ETC (Figure 1); and is embedded in the inner mitochondrial membrane where it catalyzes the transfer of electrons from reduced cytochrome c to molecular oxygen (Antonicka et al., 2003; Bohm et al., 2006). In humans, COX is a multimeric protein composed of 13 subunits with 3 encoded by the mtDNA (*MT-COX1-3*) and form the catalytic subunits of the enzyme, while the other 10 are nuclear-encoded and contribute to the assembly and biogenesis of the complex (Antonicka et al., 2003).

While isolated cases of COX deficiencies owing to mtDNA mutations have been reported (Supplementary Table 2), mutations in nuclear-encoded genes have been reported as the most common cause of Complex IV deficiency (Supplementary Table 1). Approximately 15% of LS cases worldwide are attributed to isolated COX deficiency, with mutations in the SURF1 gene accounting for at least a third of these cases (Li et al., 2018). Mutations in other nuclear genes such as *Sco1* (Mourier et al., 2014), *Sco2* (Joost et al., 2010), *Cox10* (Antonicka et al., 2003), Cox15 (Mourier et al., 2014), LRPPRC (Rolland et al., 2013; Mourier et al., 2014), *TACO1* (Weraarpachai et al., 2009), *PET100* (Lim et al., 2014), *C12orf65* (Wesolowska et al., 2015), which play important roles in COX assembly and biogenesis, have all been implicated in LS pathology.

The LRPPRC mutation is one of the most studied founder mutations, which affects the leucine-rich pentatricopeptide repeat domain protein (LRPPRC), involved in post-transcriptional regulation of mitochondrial gene expression (Olahova et al., 2015). This specific mutation is found to be prevalent and unique to the French-Canadian population in the Saguenay-Lac-Saint-Jean region of Quebec. In one of the largest known cohort patient studies, the result showed that 55 of the 56 patients were homozygous for A354V mutation in the LRPPRC gene and presented with LS or stroke-like episodes. Compared to SURF1 mutations, LRPPRC mutation resulted in a distinct occurrence of a metabolic crisis, consequently resulting in earlier and higher mortality (Mourier et al., 2014).

SURF1 mutation is the other most studied COX deficiencies resulting in LS and other metabolic disorders. Surf1 is an assembly factor of complex IV and is a member of the Surfeit locus protein localized in the mitochondrial inner membrane with multiple transmembrane domains. Although the loss of SURF1 function has been shown to result in a considerable decrease in complex IV assembly, it does not result in complete loss of the assembly, suggesting that it is important but not indispensable. A recent study reported a case of SURF1 mutation associated with LS in the Chinese population with data suggesting the presence of a mutation spectrum and the likelihood of the spectrum being population specific. The specificity of this mutation in Chinese populations related to the presence of more frameshift and nonsense mutations in this gene compared to point mutations noticed in other populations (Li et al., 2018).

Other rare mutations in assembly proteins of COX have been described. For example, eight Australian families with Lebanese ancestry diagnosed with LS or LS-like encephalopathy associated with COX deficiency had mutations in the PET100 gene (Lim et al., 2014). This mutation resulted in a similar, yet distinct clinical presentation to those of SURF1 LS from patients with Lebanese ancestry. While the exact function of this gene is still unknown, this points to PET100 as being another case of the founder’s mutation. While the SURF1 and LRPPRC seem to retain a homogenous phenotypic presentation in LS, our knowledge of the functions of these and other assembly/biogenesis proteins is still lacking. Therefore, it is still very unclear the specific role of each of these genes in the etiology of LS.

### Complex V

A multi-subunit complex, with a molecular mass of approximately 550 kDa, the mitochondrial ATP synthase, or complex V (Figure 1) is the terminal complex of the OxPhos pathway (Kucharczyk et al., 2009b). The ATP synthase has both a hydrophobic domain (F_o_), embedded in the mitochondrial inner membrane, and a hydrophilic ATPase domain (F_l_), which resides in the matrix. The mitochondrial complex V uses a proton gradient generated by the other ETC complexes (I, III, and IV) to drive the catalytic conversion of ADP (Adenosine diphosphate), and inorganic phosphate (P_i_) into ATP (Adenosine triphosphate). In humans, the complex is composed of 19 structural subunits, 2 of which are encoded by mtDNA, the other 17 encoded by nDNA. Mutations in either of the nuclear or mitochondrial encoded genes could contribute to the dysfunction of this complex, consequently resulting in decreased ATP synthesis.

Although nDNA or mtDNA mutations could affect the functions of complex V (Supplementary Table 1), mutations in the *MTATP6* gene seem to be the most prevalent pathological cause of LS related to complex V deficiency (Supplementary Table 2). The *MTATP6* gene is one of the two mtDNA genes that encode proteins forming part of the F_o_ domain of complex V, with the most common mutation being a point mutation at nucleotide position 8993. Two-point mutations in this specific nucleotide position, m.8993T > G and m.8993T > C which result in substitution of a highly conserved Leucine156 (Leu) residue for Arginine (Arg) and Proline (Pro) residues respectively have been associated with complex V deficiency (Uziel et al., 1997; Baracca et al., 2007; Debray et al., 2007). Interestingly, the m.8993T > C mutation has a milder diseased phenotype compared to the m.8993T > G mutation. The m.8993T > C mutation, even at a very high mutant load of 94% results in mild cases of NARP while the m.8993T > G result in more severe cases of Maternally Inherited Leigh Syndrome (MILS) (Santorelli et al., 1993). Furthermore, the m.8993T > C mutation has been reported to result in late-onset manifestation and slower disease progression. The relative severity of m.8993T > G compared to m.8993T > C could potentially be attributed to the amino acid substitutions of each mutation. The m.8993T > C mutation results in non-polar amino acid, Leu being substituted with another non-polar amino acid, Pro; while the m.8993T > G mutation results in substitution of non-polar amino acid for a charged (basic) amino acid, Arg. The change in polarity and introduction of a charged amino acid could result in greater destabilization in the catalytic site of the ATP synthase where this mutation occurs (Kucharczyk et al., 2009a). Further, increased ROS and Superoxide Dismutase (SOD) production by m.8993T > C mutants and significantly higher membrane potential in m.8993T > G mutants could serve as compensatory mechanisms employed in these mutations, thus serving to explain the varying phenotypes observed between the two mutations (Baracca et al., 2007). However, other research groups have reported that while both mutations affect cellular energetics and result in varying diseased phenotypes, ATP-synthesis in cells with either of these mutations is not extremely diminished (Pallotti et al., 2004; Baracca et al., 2007). These observations demonstrate that the bioenergetic defect is unlikely to be the main reason for disease pathogenesis with the need for more studies to elucidate the mechanism of disease pathology associated with these mutations.

Another mutation at nucleotide position 9185 of the *MTATP6* gene has been reported to be involved in LS. The m.9185T > C results in a substitution of Leu220 for proline and is believed to interfere with the proton pump. Similar to the m.8993 mutations, lymphoblast studies did not show abnormality in ATP synthesis. The reason for the onset of LS in individuals carrying this mutation is unknown; however, disease onset and exacerbation in m.9185T > C cases correspond to febrile viral-like illness or infection (Saneto and Singh, 2010; Piekutowska-Abramczuk et al., 2018b). In one case, hypoxia treatment reversed LS in a patient carrying this disorder while the brother of this patient with 100% mutated mtDNA for m.9185T > C mutation did not present with NARP/MILS (Piekutowska-Abramczuk et al., 2018b). In this specific case, it was postulated that hypocapnia and respiratory alkalosis as a result of hyperventilation could potentially contribute to LS and hypoxia treatment was a recommended therapeutic option to reverse disease symptoms (Piekutowska-Abramczuk et al., 2018b). However, these studies could not fully elucidate the mechanism of LS disease pathology due to the very small sample size. Therefore, more extensive studies need to be performed to better understand the role of this mutation in disease etiology.

### Other Deficiencies Involved in LS

Besides deficiencies in the ETC and ATP synthase enzymes, pathogenic mutations in gene encoding proteins essential for the maintenance of the integrity of the mitochondria have also been reported to cause LS (Lebon et al., 2007; Ghezzi et al., 2009; Fassone et al., 2010; Debray et al., 2011; Gerards et al., 2013; Baertling et al., 2017; Smith et al., 2018). Genes encoding proteins needed for assembly of OXPHOS enzymes, expression, and maintenance of mtDNA, cofactor biosynthesis, mitochondrial quality control and dynamics, and pyruvate dehydrogenase, and vitamin transport have all been implicated in LS (El-Hattab et al., 1993; Rahman and Thorburn, 1993; Benit et al., 2001; de Lonlay et al., 2001; Antonicka et al., 2003; Calvo et al., 2010; Tucker et al., 2011; Patel et al., 2012; Atwal, 2014; Smith et al., 2018). Many of the genes involved in these processes are nuclear-encoded, as described previously. Likewise, mutation to any of the mtDNA genes encoding the rRNAs and tRNAs involved in protein synthesis in the mitochondria can have a dire effect on the structural and functional integrity of the mitochondria.

Historically, the mutation in a subunit of the pyruvate dehydrogenase complex (PDHc) was one of the first observations recorded in cases of LS patients (Devivo et al., 1979). In a review of 371 patients with a spectrum of PDHc deficiency, LS was described in 50 of these patients (Patel et al., 2012). The PDHc is a multi-subunit enzyme that is involved in the conversion of pyruvate into acetyl-CoA, an important substrate of the TCA cycle. As such it connects the glycolytic pathway with the oxidative pathway of the TCA cycle (Patel and Korotchkina, 2006). Therefore, mutations affecting any of its subunits can result in disruption to mitochondrial respiration and result in reliance on the glycolytic pathway for the production of ATP. Pathogenic mutations in the PDHA1 gene have been reported as the most common PDHc deficiency involved in LS (Quintana et al., 2009; Patel et al., 2012).

Aside from PDHc, errors in thiamine metabolism were also considered one of the causes of LS when it was first described. During the early years after its initial description, its resemblance to Wernicke’s encephalopathy (WE) led many to believe that LS resulted from an error in thiamine metabolism (Worsley et al., 1965; Baertling et al., 2014; Leigh et al., 2015). Many years after its first description, it was noted that some patients with LS presented with deficiencies in either the pyruvate decarboxylase (Evans, 1981) or pyruvate dehydrogenase complex (PDHc) (Devivo et al., 1979). This is because thiamine (vitamin B1) is a cofactor used by a lot of enzymes involved in cellular metabolism (Brown, 2014). Thiamine is transported into the cell by two transporters, THTR1 and THTR2, and gets converted in the cytosol into its active form, thiamine pyrophosphate (TPP) (Brown, 2014). TPP serves as an essential cofactor for enzymes such as transketolase, PDHc, alpha-ketoglutarate dehydrogenase, and several other enzymes involved in various metabolic pathways (Brown, 2014; Gerards et al., 2016). Mutations in TPK1 (thiamine pyrophosphokinase) (Banka et al., 2014), SLC25A19, and SLC19A3 (Gerards et al., 2013; Ortigoza-Escobar et al., 2014; Ortigoza-Escobar et al., 2016) have all been implicated as the most common cause of LS resulting from thiamine deficiency. The TPK1 is involved in conversion of thiamine into its active form in the cytosol, while SLC19A3 encodes the gene for one of the thiamine transporters (THTR2), suggesting that mutations that affect the transport or metabolism of thiamine can result in dysfunction of mitochondrial energetics.

Other mutations have been reported to be involved in LS as well. Mutations resulting in defects to mitochondrial gene expression, mutations in genes encoding translation machinery such as tRNAs and mitochondrial housekeeping have been reported to cause LS. These include mutations in the mtDNA encoded tRNA MT-T1 (Cox et al., 2012), a mutation in catalytic subunits of the DNA polymerase gamma (POLG), and several nuclear-encoded translation factors (Antonicka et al., 2003; Valente et al., 2007; Tucker et al., 2011; Ahola et al., 2014; Schwartzentruber et al., 2014). Mutations in nuclear genes encoding structural and assembly subunits (Supplementary Table 1) of ETC enzymes have also been described. The most common of these are mutations in the NDUF (Budde et al., 2000; Bugiani et al., 2004; Hoefs et al., 2008, 2011; Tuppen et al., 2010b; Uehara et al., 2014; Lou et al., 2018; Sofou et al., 2018) and SURF1 (Tiranti et al., 1999; Sonam et al., 2014; Li et al., 2018)genes. Mutations in nuclear genes resulting in CI or CIV deficiencies (Supplementary Table 1) account for a large percentage of the nuclear mutations associated with LS (Koene et al., 2012; Gerards et al., 2016). Advances in whole-genome and exome technology continue to result in the identification of isolated and multiple complex deficiencies that contribute to LS.

## Disease Models for LS

Owing to the difficulty of manipulating the mammalian mitochondrial genome and the paucity of animal models, several model organisms have been developed over the years to study mitochondrial-related disorders. Some of these models include organisms like yeasts (Srivastava et al., 2018), fruit flies (Scialo et al., 2016), and worms (Lin and Wang, 2017). Much of the structure of the mitochondrial ETC complexes were first determined through crystallization studies using yeast as the model organism (Srivastava et al., 2018). Therefore, it is important to highlight how these models have proven to be invaluable in our quest to understanding mitochondrial disorders such as LS. The following section discusses some of the breakthroughs that have been recorded using the current models. Furthermore, the limitations of each of these models are briefly discussed to accentuate the importance of developing an appropriate disease model for studies of LS and related mitochondrial disorders.

### Yeast

*Saccharomyces cerevisiae* is the most widely used organism for studying mitochondrial genetic disorders (Tuppen et al., 2010a), largely in part to the structural similarity between yeast mt-tRNAs and the possibility of transforming yeast to construct various mitochondrial mutations (Ahlers et al., 2000; Feuermann et al., 2003; Tuppen et al., 2010a). Furthermore, the ability of *S. cerevisiae* to survive in the complete absence of functional mtDNA makes it an attractive model for studying severe mitochondrial defects (Tuppen et al., 2010a; Ceccatelli Berti et al., 2021; Di Punzio et al., 2021). Yeast models have been employed in investigating various mitochondrial tRNA mutations (De Luca et al., 2006; Montanari et al., 2008) involved in MELAS (mitochondrial myopathy, encephalopathy lactic acidosis and stroke-like episodes) (Feuermann et al., 2003), MERRF (myoclonic epilepsy and ragged red fibers), CPEO (chronic progressive external ophthalmoplegia) (Rohou et al., 2001) and other related myopathies. Yeast has also been used to characterize and model the m.T8993C mutations which are a common cause of NARP and MILS (Tuppen et al., 2010a). Early studies on caloric restrictions and longevity in *S. cerevisiae* resulted in the discovery that caloric restriction extends lifespan in yeast (Jiang et al., 2000; Lin et al., 2000; Schleit et al., 2013). It was these findings that paved the way for the use of mTOR inhibitors as potential therapies for mitochondrial disease (Johnson et al., 2013; Sage-Schwaede et al., 2019; Cheema et al., 2021).

Although this model has been instrumental in better understanding various mitochondrial-related disorders, marked evolutionary distance between yeast and humans does not allow for conclusive information about the impact of these defects on tissues and organs. Furthermore, fermentative yeast such as *S. cerevisiae* lack complex I, making it impossible to model one of the most common causes of a mitochondrial disorder. The introduction of an alternative model, the obligate aerobic yeast, *Yarrowia lipolytica*, however, could potentially resolve this problem. *Y. lipolytica* possesses a vital proton-pumping NADH:ubiquinone oxidoreductase, making it possible to study the structure and function of complex I in health and disease. In this model system, three missense mutations in nuclear-coded subunits homologous to bovine TYKY (NDUFS8) and PSST (NDUFS7) of the mitochondrial complex I was developed to understand the function of these subunits (Ahlers et al., 2000); and led to conclusions that NDUFS8 and NDUFS7 might be involved in proton translocation by complex I. While this model solves the problem associated with a lack of complex I in *S. cerevisiae*, it still does not address the variation in homology and evolutionary distance between yeast and humans. Therefore, the yeast model is still not able to answer all the questions regarding mechanisms of disease pathology in various mitochondrial-related disorders.

### Worm

The nematode, *Caenorhabditis elegans* (*C. elegans*) is another model organism that has been used for studying LS. *C. elegans* are simple multicellular organisms with only 959 cells, organized into gastrointestinal, reproductive, muscle, nerve, and cuticle tissues. The genome of *C. elegans* has been fully sequenced and it is known to share greater than 83% homology with the human genome (Lai et al., 2000; Maglioni et al., 2020). The transparency of the worm and its small size makes it easier to directly visualize cellular processes and manipulate their genes to model various human diseases (Kuwabara and O’Neil, 2001; Maglioni et al., 2020). Furthermore, the complete outline of the neurons and synaptic connectivities in *C. elegans* have been determined, making it ideal for studying neurodegenerative disorders like LS (Riddle and Albert, 1997; Maglioni et al., 2020). It has been used as a model to study neuronal alterations during aging and as a model for neurodegenerative diseases like Alzheimer’s and Parkinson’s disease (Lublin and Link, 2013; Alexander et al., 2014). Disease models of LS with human homologs of NDUFS4 and NDUFS1 mutations affecting CI were created with *C. elegans* to screen for drugs that can suppress the disease (Maglioni et al., 2020). Not only does the model in this study recapitulate the human pathologies associated with the mutations, but they also led to the discovery that the mutations were causing alterations in acetylcholine synapsis. In another study, a human homolog of the NDUFS2 mutant model was created to study the preventative and therapeutic effects of various antioxidants that have been proposed for the clinical treatment of respiratory chain diseases (Polyak et al., 2018). Human homologs with missense and deletion mutations have also been created for genes such as *SDH-C* (CII), *UQCRFS1* (CIII), *COQ7*, and *IDH1* (Lai et al., 2000). These mutant strains have allowed for modeling respiratory chain dysfunction and investigating *in vivo* mitochondrial functions associated with these defects (Kayser et al., 2004; Lemire et al., 2009; Dingley et al., 2010). These types of studies where mutations are introduced to study disease phenotypes while screening for targeted therapeutics continue to provide valuable information on mechanisms underlying the mutation variants involved in LS and other respiratory chain disorders (Diaz, 2010; Polyak et al., 2018; Maglioni et al., 2020; Fox et al., 2021).

While worm models present several advantages such as short lifecycle/lifespan, the relative ease and low cost of generating transgenic strains, and their transparent nature which allows for cellular localization of fluorescently tagged genes, they present with their limitations (Kamath et al., 2001; Timmons et al., 2001; Rea et al., 2010), One of the major challenges of using worms as disease models is in the ability to obtain evidence that observed pathologies are specific and relevant to the disease being studied (Teschendorf and Link, 2009). The evolutionarily conserved genes between worms and humans make it easy to introduce mutations to replicate the involvement of nDNA in mitochondrial disorders. However, mtDNA mutations have a mild effect in *C. elegans* and do not mimic the features associated with these pathogenic variants in humans (Tsang and Lemire, 2003). Since mtDNA mutations can also result in LS, worms might not be ideal models for studying pathophysiological features associated with mutations in the mitochondrial genome. Nevertheless, worm models recapitulate many key features of proteins of interest in human respiratory chain disorders and continue to serve as critical foundations for furthering our understanding of these disorders.

### Fruit Fly

Given that neurodegeneration is one of the hallmarks of LS, the fruit fly, *Drosophila melanogaster*, has proven to be invaluable in understanding the cellular, and molecular genetic mechanisms underlying neurodegeneration (reviewed in Deal and Yamamoto, 2018). Studies with *Drosophila* have allowed the identification of certain evolutionarily conserved genes that contribute to neurodegeneration in humans. Furthermore, the fruit fly has been used to construct models of various LS mutations. Transcriptional silencing of CG9943, the Drosophila homolog of SURF1 resulted in decreased COX activity, mirroring some of the same ETC defects observed in humans with SURF1 mutations (Da-Re et al., 2014). In addition to dysregulation of COX activity, it was also observed that activity of the F_o_-subunit of the ATP synthase and the other OXPHOS complexes were affected. This suggests an additional role in the organization of OXPHOS complexes for the SURF1 gene in addition to its role in the assembly of COX (Da-Re et al., 2014). Knockdown in another *D. melanogaster* gene, ND-18, an ortholog of the human NDUFS4 recapitulates the feeding difficulty observed in humans with a defect in this complex I gene (Foriel et al., 2018). One of the biggest advantages the fruit fly model offers is that knockdown models reduce the impact of heteroplasmy, allowing for recapitulation of key features of LS disorder in humans and enabling detailed *in vivo* studies of LS and other associated mitochondrial disorders (Sanchez-Martinez et al., 2006; Foriel et al., 2018; Loewen and Ganetzky, 2018). Although *D. melanogaster* continues to be used as a model to study the effects of mutations on mitochondrial functions and the consequent effect on neurodegeneration (Chen and Feany, 2005; Jeibmann and Paulus, 2009; Chen et al., 2016), the anatomical divergence between humans and fruit fly limits recapitulation of certain morphological features. While the fundamental molecular pathways might be conserved, these differences possess limitations in the *D. melanogaster* models; thus requiring significant follow-up studies in higher model organisms of any genes observed as involved in LS pathology in *D. melanogaster*.

### Mouse

Although yeast, worms, and fruit fly models have been invaluable in advancing our understanding of mitochondrial genetics, a mammalian *in vivo* model system is required to fully comprehend the etiology, pathogenesis, and tissue specificity associated with mitochondrial diseases. Where the yeast, worms and, fruit fly models lack, the mouse model is ideal largely in part to its evolutionary closeness to humans. Advances in gene targeting have revolutionized life sciences and made it possible to generate mouse models that can recapitulate mitochondrial diseases (Vempati et al., 2008), with mouse models of OXPHOS defects (extensively reviewed by Torraco et al., 2009) proving to be invaluable tools in furthering our understanding of various mitochondrial diseases. Transmitochondrial mice models developed to study mutation selectivity in female mouse germline introduced with two different mutations; a severe ND6 and milder CO1 mutations using a heteroplasmic mice model, showed that while some tissues show random genetic drift in their mtDNA; in other tissues, there seems to be a strong, tissue-specific, age-related directional selection for different mt-DNA genotypes even in the same animals (Jenuth et al., 1997; Fan et al., 2008). Few studies have also developed an LS model for neuroprotection studies by using the complex I inhibitor MPTP (1-methyl-4-phenyl-1,2,3,6-tetrahydropyridine). MPTP intoxicated mice served as a good model of LS based on the finding that MPTP affects mainly complex I and complex IV and triggered basal ganglia degeneration (Lagrue et al., 2009; Da Costa et al., 2016). Mice missing the NDUFS4 subunit of complex I are the leading models for LS (Quintana et al., 2010, 2012; Adjobo-Hermans et al., 2020; Grillo et al., 2021). Studies on Ndufs4 knockout (KO) led to the discovery that hypoxia can prevent and reverse neurological effects of CI-deficiency and LS (Ferrari et al., 2017; Jain et al., 2019). These studies revealed that hypoxia extended the lifespan in KO mice by up to 4 times those in normoxic conditions (Ferrari et al., 2017). When KO mice with late-onset encephalopathy were exposed to normobaric 11% O_2_, the neurological disease in these mice improved. Once they were returned to normoxia, the Ndufs4 KO mice died within days (Ferrari et al., 2017), suggesting that intermittent hypoxia was ineffective in preventing neuropathology. A similar result was observed in another study, where activation of the hypoxia pathway was not sufficient to rescue disease in the Ndufs4 KO mouse model. However, chronic hypoxic breathing and other intervention to reduce brain oxygen levels were effective at preventing neurological disease in these mouse models (Jain et al., 2019). These studies attributed a role for unused oxygen in the pathology observed under normoxic and intermittent hypoxic conditions (Ferrari et al., 2017; Jain et al., 2019). The Ndufs4 KO has been beneficial in understanding the potential for hypoxia as a therapeutic strategy for LS. Another study with Ndufs4 KO showed that not only did the mutation result in a significant decrease in CI subunit levels; it also induced a near-complete loss of another accessory subunit, NDUFA12. The mutation also resulted in a significant increase in a different assembly subunit NDUFAF2, leading to the conclusion that NDUFAF12 could stabilize CI in the absence of NDUFS4 and NDUFA12 (Adjobo-Hermans et al., 2020). In addition to the Ndufs4 KO, mouse models have been engineered for studying deficiencies in other ETC complexes that contribute to LS. Homozygous Surf1 mutant mice with cytochrome c oxidase deficiency (Agostino et al., 2003), mice lacking mitochondrial superoxide dismutase (Melov et al., 1998), and mice deficient in the mitochondrial membrane protease Presenilins-associated rhomboid-like protein (PARL) (Spinazzi et al., 2019) have all been created to model LS.

Whilst the mouse model has been invaluable in answering many questions associated with mitochondrial disease etiology and pathogenesis, it seems to pose some challenges just like the other models described previously. One of the biggest setbacks involves overcoming problems of embryonic and neonatal lethality of mutant mice to examine if the mice faithfully reproduce targeted disease as expected (Vempati et al., 2008). Nevertheless, mouse models have been beneficial in overcoming challenges associated with the availability of human samples to conduct comprehensive mechanistic analysis and continue to be used as a model for *in vivo* studies of mitochondrial diseases.

### Transmitochondrial Cybrids

After recognition in 1963 that the mitochondria contain their own genome and the realization that under conditions favoring glycolytic metabolism, yeast will undergo depletion and deletion of mtDNA molecules, efforts were made by scientists to artificially induce this phenomenon using ethidium bromide (Swerdlow, 2007). It was not until 1985 that the first report of a total eukaryotic cell mtDNA depletion was reported in a chicken embryo (Desjardins et al., 1985; Morais et al., 1988). In 1989, King and Attardi reported the successful production of human osteosarcoma rho zero (ρ^o^) cell lines; a significant progress that gave rise to the development of human transmitochondrial cytoplasmic hybrid (cybrids) models for the investigation of mitochondrial related diseases (King and Attardi, 1989). This model is especially appealing because it delineates donor mtDNA from the original nuclear background, allowing for the study of mitochondrial mutation-dependent differences in isolation (Danielson et al., 2005).

Transmitochondrial cybrids are generated by a cytoplasmic fusion of ρ^o^ cells from parent cell lines with enucleated cells from donor cell lines and has allowed for studies on the heteroplasmic threshold, mtDNA-nDNA compatibility (Swerdlow, 2007), mtDNA segregation (Jenuth et al., 1996, 1997), and most importantly for studying various disorders of the mitochondria such as LHON (Danielson et al., 2005; Iyer, 2013) and LS (Iyer et al., 2009b, 2012). Transmitochondrial cybrid models have also resulted in questioning the uneven segregation hypothesis that has been used in explaining the genetic-phenotypic variance associated with various mitochondrial diseases (Rorbach et al., 2008). While this model provides an attractive option, it poses some challenges; one of the most obvious being the technique used in generating the cell lines. The ρ^o^ cells are generated through long-term exposure to a mutagen, ethidium bromide, while the enucleated donor cells are generated by exposure to cytochalasin B; a mycotoxin (Danielson et al., 2005), a process that could induce significant cellular stress and affect several gene expressions. Furthermore, the fusion process may result in damage and disorganization of multiple cellular organelles and membranes. Another challenge results from the use of aneuploid inherently genetically unstable cancer cell lines as the parental cell lines used in this model (Danielson et al., 2005) as this could potentially result in dysfunctional or variable gene expression. Finally, given that we do not fully understand the cross-talk that exists between nuclear and mitochondrial DNA, a cybrid cell might not fully recapitulate the disease of interest.

### Induced Pluripotent Stem Cell (iPSCs) Models

The discovery of induced pluripotent stem cells (iPSCs) by Yamanaka in 2006 opened a plethora of opportunities for biomedical research. Not only does this technology allow for the reprogramming of differentiated cells to an embryonic-like state, it also opened the possibilities for a new era of personalized medicine; allowing for the generation of patient-specific cell lines for the study of various diseases (Takahashi et al., 2007). This technology is especially useful in investigating mitochondrial diseases owing to their varying phenotypes. One of the challenges associated with understanding the pathogenesis of mitochondrial diseases is the limited availability of human samples and variability in diseased phenotypes between and within tissues. With iPSCs, some of these problems can be resolved as fibroblast derived from diseased patients could be reprogrammed and potentially differentiated into any cell/tissue to better elucidate the pathological mechanism of a specific mitochondrial disorder on various cells/tissues. Many recent studies have demonstrated the potential for generating human iPSC patient-specific models from LS carrying mutations in MT-ATP6 and MT-ND5 genes (Galera-Monge et al., 2016; Zurita-Diaz et al., 2016; Grace et al., 2019). Another study combined somatic cell nuclear transfer (SCNT) and iPSC technology to perform a metabolic rescue in the iPSCs generated from these patients, demonstrating the significant possibilities that iPSC models hold, as they could potentially be used to simultaneously study pathogenesis and develop treatments for patients with LS and other mitochondrial diseases (Ma et al., 2015). With unique hiPSC model systems that carry defined mtDNA mutations, the potential for a better understanding of key pathways and metabolic regulation during the early development of LS is now possible. The generation of patient-specific hiPSCs and subsequent generation of specialized differentiated cell types also allows us to better understand the variability associated with LS and the development of targeted therapies. In addition, it now allows us to address the extent to which heteroplasmy is preserved in specific cell types during the differentiation process.

One of the challenges with iPSC models is the ability to maintain mutation load in daughter cells after reprogramming and differentiation (Povea-Cabello et al., 2020). To address this problem, direct reprogramming protocols have been developed to ensure that the age and epigenetic markers of donors are maintained (Horvath, 2013; Mertens et al., 2015; Huh et al., 2016). Another challenge is associated with the conversion efficiency and generation of high purity samples that only contain the cell lineages of interest. Recent studies with induced neurons (iN) have developed reprogramming techniques that address this problem, resulting in a high yield and percentage of pure iNs (Drouin-Ouellet et al., 2017; Shrigley et al., 2018). The biggest challenge of all relates to maintaining these cell lines in long-term culture. The long-term culture of iPSC and reprogramming is difficult and extremely expensive, making it difficult to adopt in labs with limited resources (Povea-Cabello et al., 2020). However, direct reprogramming, like those done to derive iNs can help reduce the cost of reprogramming hiPSC. Recently, iNs from two MERRF patient-derived fibroblasts harboring the m.8344A > G were successfully developed using direct reprogramming (Villanueva-Paz et al., 2019). These iNs retained the heteroplasmy mutation load from the parent fibroblast and showed features of matured neurons. Neuronal maturation markers and functional studies such as electrophysiological recordings further confirmed that these iNs behaved like neurons. Furthermore, the MERRF iNs showed pathophysiological features that have been described in other MERRF models, making them suitable models for mimicking neurological disorders (Villanueva-Paz et al., 2020). Together, hiPSC and directed reprogramming technology hold promise for advancing our understanding of mitochondrial disorders like LS, while allowing for targeted high-throughput drug screening in human cell lines and advancing precision medicine (Villalon-Garcia et al., 2020). This is by far the most accurate model because the same cell line from a single patient has the potential to be differentiated into any cell type to study the effects of pathogenic mutations on the different cells and tissues in humans. Drug screening using this technology could allow for quicker discoveries because it eliminates the need of testing these drugs in other animal models.

## Therapeutic Strategies and Future Directions

Despite the progress that has been made in understanding the molecular mechanisms underlying mitochondrial diseases, there are currently no specific treatment options for LS and other related mitochondrial disorders. Currently, the available therapeutic approaches are limited and are still an area of interest for various research groups. The currently available options are symptomatic treatments; and focus on improving energy state through optimization of ATP production while lowering lactate levels (Ruhoy and Saneto, 2014). EPI-743, an analog of coenzyme Q10, is an antioxidant that has been suggested to improve clinical outcomes in some cases of genetically confirmed LS in a small controlled study (Martinelli et al., 2012), while supplementation with B vitamins such as thiamine, a cofactor of PDH (pyruvate dehydrogenase) is being considered as a treatment option (Bar-Meir et al., 2001). The ketogenic diet has also been proposed to improve symptoms in an adult case of LS (Malojcic et al., 2004) and some studies have also suggested hypoxia treatment as a therapy for mitochondrial disease (Russell et al., 2016; Shoubridge, 2016). While this might seem counterintuitive, studies in mice suggest that hypoxia could potentially be used as a therapy for mitochondrial dysfunction, with the researchers proposing that hypoxia could be beneficial owing to two different mechanisms: the first involving a reduction in O_2_ tension, resulting in a decrease of reactive oxygen species (ROS), while the second involving activation of HIFs (Hypoxia-Inducible Factors), resulting in decreased production of ATP by OXPHOS and a shift to glycolysis (Jain et al., 2016, 2019; Ferrari et al., 2017). Extensive research needs to be performed to validate the mechanism(s) involved in this process before hypoxia is deemed safe/efficient as a treatment for any mitochondrial disorder. Furthermore, the efficacy and safety of many of the supplements and vitamins are still being investigated. Many of these currently lack valid preclinical and clinical evidence to support their efficacy in treating mitochondrial disorders (Garone and Viscomi, 2018). One of the biggest challenges to developing treatment options for LS is the phenotypic and genotypic variability associated with this mitochondrial disorder. Nevertheless, efforts are being made to develop a novel treatment, with efforts focusing on therapies to increase ATP production by the ETC, increasing mitochondrial biogenesis, targeting dysfunctional mitochondria for degradation, or degrading mitochondrial genomes harboring disease-causing mutations. The collective goal of these treatment options is to improve mitochondrial health in specific cells and tissues that are impacted by the disease.

### Mitochondrial Replacement Therapy

Since mtDNA are maternally inherited and mutations in the mt genome can result in a range of pathologies and there is currently no cure for these diseases, mitochondrial replacement therapies have gained attention as a means of limiting inheritance of pathogenic mtDNA (Kang et al., 2016; Greenfield et al., 2017; Saxena et al., 2018; Craven et al., 2020). Two techniques, one developed in the United Kingdom and the other in the United States, have been established to prevent the transmission of mutations in mtDNA (Saxena et al., 2018; Craven et al., 2020). In the United Kingdom, the method that was developed and currently licensed involves pronuclear transplantation (PNT) from an affected donor into an enucleated healthy embryo right after completing meiosis. Reports from preclinical studies using this technique showed that mtDNA carryover was reduced to <2% in 79% of pronuclear transfer (PNT) blastocysts (Hyslop et al., 2016). In the United States, the approach developed is based on maternal spindle transfer (Kang et al., 2016; Zhang et al., 2017), and involves the transfer of the nucleus of the affected mother’s oocyte to an enucleated donor’s oocyte before fertilization with the father’s sperm. The maternal spindle transfer resulted in embryos containing >99% of donor mtDNA. While donor mtDNA was stably maintained in embryonic stem cells (ES cells) derived from most embryos, some of the ES cell lines reversed to the maternal haplotype (Kang et al., 2016), leading to the suggestion that some haplotypes confer proliferative and growth advantages to cells. Therefore, having leftover mtDNA from the affected mother could still result in the development of the mitochondrial disorder in the offspring later on.

In the United States, the first baby born by the spindle transfer technique generated a lot of controversies (Zhang et al., 2017; Garone and Viscomi, 2018). There are still so many ethical issues surrounding the use of mitochondrial replacement therapy (Craven et al., 2020). This is evident in the fact that there are very few countries that have adopted this. Even in countries like the United Kingdom where its use has been approved, the approval is only for selected patients (Garone and Viscomi, 2018; Craven et al., 2020). Aside from the ethical dilemma, both techniques that have been developed still have a small fraction of the affected mother’s mtDNA present (Hyslop et al., 2016; Kang et al., 2016; Zhang et al., 2017). As reported in one of the studies, there was a gradual loss of donor mtDNA in some of the ES cells derived from transplanted embryos. Long-term follow-up on children born because of mitochondrial replacement therapy would help clarify if they develop mitochondrial disorders later in life. Another concern that has been raised relates to mitochondrial-nuclear DNA compatibility. However, studies have suggested that switching nuclear genomes between different mitochondrial haplotypes does not result in any detectable difference in mitochondrial gene expression (reviewed extensively by Greenfield et al., 2017). The other limitation is that mitochondrial replacement therapy only addresses mitochondrial disorders resulting from a mutation in the mitochondrial genome, but not the nuclear genome.

### Gene Therapy

Gene therapy promises to use developments made from gene-editing technologies to cure mitochondrial diseases. Since mutations in mtDNA contribute to mitochondrial disorders, editing these mutant DNA shift the proportions of mutant to healthy DNA, thereby, reducing the burden of the disease. Currently, restriction endonucleases (RE), zinc finger nuclease (ZFN), transcription activator-like effector nuclease (TALEN), and CRISPR/Cas9 technology are widely used editing tools for this purpose (Greenfield et al., 2017; Reddy et al., 2020; Yang et al., 2021). The RE, Smal which is usually used for diagnosis of NARP and LS because of its ability to recognize the DNA sequence in the pathogenic variant of the m.8993T > G mutation was modified to eliminate this mutant mtDNA. In this study, elimination of this pathogenic mtDNA was followed by repopulation of wild-type mtDNA and restoration of mitochondrial functions (Tanaka et al., 2002). Studies using ZFN (Minczuk et al., 2008; Gammage et al., 2014, 2016, 2018) and TALENS (Bacman et al., 2013; Reddy et al., 2015; Yang et al., 2018) have also been used to successfully target pathogenic variants in the mitochondrial genome to eliminate these mutant genes and restore wild-type phenotype. Although a lot of these techniques have been readily adapted to editing the nuclear genome, slight modifications have to be made to target them to mitochondria to modify mtDNA (Hussain et al., 2021). For instance, while CRISPR/Cas9 has been adopted for base editing in the nuclear genome, it has been challenging to do the same with the mitochondrial genome because of the difficulty associated with the delivery of guide RNA into the mitochondria (Greenfield et al., 2017; Hussain et al., 2021; Yang et al., 2021). However, a CRISPR-free technology involving the use of bacterial cytidine deaminase toxin has been recently developed for use in mitochondrial base editing (Mok et al., 2020). This new technology has the potential to pave the way for new studies involving the precise manipulation of mtDNA to treat mitochondrial disorders.

### Pharmacological Treatments and Diet

The pharmacological therapies focus on different aspects of mitochondrial function, ranging from upregulation of mitochondrial biogenesis or autophagy to preventing oxidative damage (Lightowlers et al., 2015; Gerards et al., 2016; Povea-Cabello et al., 2020). The most common targets for pharmacological agents focus on AMP-activated protein kinase (AMPK), Sirtuins (Sirt1), and mammalian target of rapamycin complex 1 (mTORC1) pathways (Garone and Viscomi, 2018; Viscomi and Zeviani, 2020). The peroxisomal proliferator-activated receptor-gamma 1 (PGC1α) is a transcriptional coactivator for several transcription factors. Post-translational modification by AMPK or deacetylation by Sirt1 results in activation of PGC1α (Viscomi and Zeviani, 2020). Therefore, pharmacological modulations on AMPK and Sirt1 are used to activate PGC1α to enhance mitochondrial biogenesis. Since AMP usually activates AMPK, while Sirt1 is activated by NAD+, analogs of AMP and NAD + are used to activate this pathway. Compounds such as bezafibrate, AICAR, resveratrol, and nicotinamide riboside are examples of AMP and NAD + analogs that have shown some success in treating mitochondrial disorders (Bastin et al., 2008; Wenz et al., 2008; Cerutti et al., 2014; Iannetti et al., 2018). The other pathway that is targeted involves the mTORC1, which is a cytosolic Serine/Threonine kinase belonging to the phosphatidylinositol kinase-related protein kinases family. It plays a central role in processes such as protein translation, immune response, nucleotide and lipid synthesis, glucose metabolism, autophagy, and lysosomal biogenesis (Saxton and Sabatini, 2017). As discussed previously, studies on caloric restriction in yeast led to the idea that inhibition of mTORC1 can be used as therapy for mitochondrial disease. Rapamycin and its analogs are potent inhibitors of mTORC1 and have been shown to improve symptoms in some patients with mitochondrial disease (Sage-Schwaede et al., 2019; Martin-Perez et al., 2020). The effect of rapamycin has been tested in NDUFS4 KO mice, with results from this and other studies showing that mTORC1 inhibition alleviated mitochondrial disease in the mouse model of LS (Johnson et al., 2013; Cheema et al., 2021). It has been suggested that rapamycin acts by inducing a metabolic shift from glycolysis to amino acid metabolism, reducing the buildup of glycolytic intermediates (Schleit et al., 2013).

In line with the idea of metabolic shift is the ketogenic diet. This type of diet aims to shift metabolism toward beta-oxidation and ketone body production, to increase transcription of OXPHOS, TCA cycle, and glycolysis genes (Bough et al., 2006). In mouse models following a ketogenic diet, a decrease in mitochondrial ROS, and an increase in mitochondrial uncoupling protein and glutathione levels were reported (Sullivan et al., 2004; Jarrett et al., 2008), suggesting that ketogenic diets might act to reduce mitochondrial-mediated oxidative stress. However, there is still a lot of controversy regarding the safety and efficacy of this dietary option (Garone and Viscomi, 2018; Kuszak et al., 2018).

Other compounds in use are antioxidants such as CoQ10 and its derivatives idebenone, and EPI-743. Idebenone is taken up more readily by the cells and has been suggested as a replacement for CoQ10 (Kwong et al., 2002). Although idebenone has been used mainly as a treatment for Leber’s hereditary optic neuropathy (LHON), it is now being tested as a treatment option for LS (Haginoya et al., 2009; Carelli et al., 2011; Barboni et al., 2013). EPI-743 has also shown some promise in reversing LS in patients with different mtDNA mutations; however, its efficiency is still being evaluated (Gerards et al., 2016). Recently, cell permeant ETC substrates have gained attention as therapeutic agents for mitochondrial diseases. Supplementation with the cell-permeable dimethyl ketoglutarate (DMKG) extended life and delayed onset of neurological phenotype in an Ndufs4-KO mouse model of LS (Lee C. F. et al., 2019). Cell permeant succinate prodrugs have also shown promise in alleviating disease in cellular models of mitochondrial disorders with complex I deficiency (Ehinger et al., 2016; Janowska et al., 2020; Piel et al., 2020). These substrates work by increasing TCA cycle intermediates and providing alternative substrate sources for the mitochondria. Many of the pharmacological agents mentioned in this section are still in pre-clinical and clinical trials, with some of them showing conflicting results in these trials (Garone and Viscomi, 2018). Therefore, a more extensive study needs to be performed to determine the efficacy and safety of these therapeutic agents.

### Hypoxia

During aerobic respiration, oxygen acts as the final electron acceptor in the ETC, in a reaction that result in its reduction to water. In excess, oxygen can also result in the formation of reactive oxygen species (ROS) and other radicals that can damage proteins, nucleic acids, phospholipids, and other molecular species (Viscomi and Zeviani, 2019). Physiologic levels of ROS act as signaling molecules to regulate key mitochondrial functions (Butow and Avadhani, 2004). However, too much ROS can be detrimental to cells as previously highlighted. It is for this reason that cells have devised ways of neutralizing the debilitating effects of ROS and other free radicals with ROS scavengers and antioxidants. Mitochondrial disorders are problematic because they cause defects in mitochondrial ETC, compromising the efficient utilization of oxygen and electron transport. This could result in electron leakage and further result in the production of ROS and other free radicals (Viscomi and Zeviani, 2019). It is this understanding that has led to the use of antioxidants as therapeutic agents for mitochondrial disorders. However, reducing oxygen below a certain threshold, as in the case of hypoxia can be detrimental to cellular respiration as well. This is why the use of hypoxia as therapy might seem counterintuitive as a treatment for diseases wherein ETC activities are already compromised.

The use of hypoxia as a therapy for mitochondrial disorders became popular when CRISPR/Cas9 screening identified the Von Hippel-Lindau (VHL) factor as the most effective suppressor of antimycin-induced mitochondrial dysfunction (Jain et al., 2016). VHL is a ubiquitin ligase that targets hypoxia-induced transcription factors (HIFs) for degradation (Garone and Viscomi, 2018; Viscomi and Zeviani, 2019). However, hypoxia stabilizes HIF and results in the activation of hypoxia response. In a 30 day exposure to chronic hypoxic conditions (11% O_2_) Ndufs4 KO mice showed drastic improvement in lifespan and delay in clinical progression of LS. When mice with the same KO mutation were exposed to hyperoxic conditions (50% O_2_), their conditions worsened. Further, when exposed to intermediate levels of hypoxia (17%), there was no beneficial effect seen in the Ndufs4 KO mice (Jain et al., 2016). Interestingly, transcriptional activation of HIF in Ndufs4 KO mice did not result in the same beneficial effects observed under hypoxic conditions in the previous study (Jain et al., 2019). In the study, the researchers suggest that interventions to reduce brain oxygen levels are effective at preventing neurological disease in mouse models. One of the biggest challenges with the hypoxia treatment is that most studies have only been performed in the Ndufs4 KO mouse models (Jain et al., 2016; Ferrari et al., 2017; Grange et al., 2021). Studies on models with other mutations involved in LS will provide more insight into the efficacy of this therapy. Furthermore, the mechanism behind the observed response to hypoxia is still unclear and more studies need to be done to explore this (Garone and Viscomi, 2018; Viscomi and Zeviani, 2019). Finally, the methods that have been proposed in this study to lower pO_2_ are unlikely to be transferred for use in patients that are already severely sick (Jain et al., 2019).

## Conclusion

While advances in mitochondrial genetics and structural analysis coupled with sequencing have furthered our understanding of LS, observations that bioenergetic defects might not be the sole explanation for the pathology observed in LS merits further investigation (Kucharczyk et al., 2009b). Another unanswered question stems from the role of assembly/accessory factors in disease pathology. As discussed earlier, novel pathogenic roles for assembly and biogenesis factors of the ETC are being reported. Although previous studies largely focused on mutations affecting structural subunits of the ETC, we are now starting to observe that more than just the structural unit, the assembly factors, also play key roles in regulating bioenergetics of the cell (D’Aurelio et al., 2010; Pitceathly et al., 2013). Future studies thus need to be performed to understand the roles of the accessory subunits of each of the various complexes of the ETC. In addition, future research also needs to focus on mtDNA segregation in tissues, due to genetic drift in mtDNA segregation, and observations show that this drift is tissue-specific. Therefore, a better understanding of this issue could potentially provide a better understanding of the genotype-phenotype variability associated with LS and other mitochondrial diseases. Finally, the mitochondria are dynamic organelles that constantly undergo rounds of fission and fusion to maintain a healthy pool and sustain energy production. There is thus significant interest in understanding how the dynamic nature of this organelle contributes to health and disease. As outlined above, there is still much to learn about the mitochondria, as new tools and technology continue to develop, and our understanding is further enhanced. hiPSC technology is a novel tool that would allow us to bypass the paucity of human samples and provide the amount of material required to aid with studies on mitochondria. Furthermore, in combination with various gene-editing tools, hiPSC technology could potentially open doors for new drug discovery and therapeutic approaches that could invariably lead to the discovery of treatments for patients with LS.

## Author Contributions

ABB, EJL, and SI contributed to writing the manuscript. All authors contributed to the article and approved the submitted version.

## Conflict of Interest

The authors declare that the research was conducted in the absence of any commercial or financial relationships that could be construed as a potential conflict of interest.

## Publisher’s Note

All claims expressed in this article are solely those of the authors and do not necessarily represent those of their affiliated organizations, or those of the publisher, the editors and the reviewers. Any product that may be evaluated in this article, or claim that may be made by its manufacturer, is not guaranteed or endorsed by the publisher.

## Supplementary Material

Supplementary Table 1 Nuclear genes involved in LS and LS-like disorders.

Supplementary Table 2 Mitochondrial genes involved in LS and LS-like disorders.
