# Supplementary material for: Leigh Syndrome: A Tale of Two Genomes
Source: Front Physiol. 2021 Aug 11;12:693734. doi: 10.3389/fphys.2021.693734 (PMC8385445; doi:10.3389/fphys.2021.693734)
Supplement: Supplementary Table 1 — Nuclear genes involved in LS and LS-like disorders. [file Table_1.docx]

**Supplementary Table 1. Table showing nuclear genes involved in LS and LS-like disorders.** The most prevalent mutations affecting the different ETC complexes are summarized with the genetic defect and corresponding biochemical and clinical manifestations.

| **Nuclear genes involved in LS and LS-like disorders** | | | |  | |  | |  | |  |  |
| --- | --- | --- | --- | --- | --- | --- | --- | --- | --- | --- | --- |
| **Complexes** | **Structural/**  **Assembly** | **Genes affected**  **in LS** | **Genetic**  **defect** | | **Biochemical defect** | | **Clinical manifestations** | | **References** | | |
| **CI** | Structural | NDUFA1 | c.55C>T | | Elevated blood/CSF lactate and pyruvate levels, isolated CI deficiency and decreased amount of fully assembled enzyme, depolarization of MMP, decreased carnitine, elevated alanine levels, hypoglycemia close to death, decreased CIII activity, elevated cytochrome c oxidase and citrate synthase activity, decreased pyruvate oxidation (normal succinate oxidation), increase CII-CIV activity, elevated CSF protein levels, Isolated PDHs activity in skeletal/cardiac muscle, brain, liver and skin fibroblast, elevated levels of fumarate and malate | | Pyramidal tract dysfunction, ataxia, signs of brain stem dysfunction, oculomotor abnormalities, seizures, and lethargy, leukoencephalopathy, muscular hypotonia, myoclonic epilepsy, progressive macrocystic leukoencephalopathy with brain atrophy, and subsequent apnea, early-onset ophthalmoplegia, lethal encephalopathy | |  | | |
|  | Structural | NDUFA2 | c.208+5G>A, c.875T>C, c.866+4A>G | |  |  |  |  | (Uehara et al., 2014)  (Hoefs et al., 2008;Tuppen et al., 2010b;Schlehe et al., 2013) | | |
|  | Structural | NDUFA9 | c.962G>C | |  |  |  |  | (van den Bosch et al., 2012) | | |
|  | Structural | NDUFA10 | c.1A>G, c.425A>G | |  |  |  |  | (Hoefs et al., 2011) | | |
|  | Structural | NDUFA12 | c.178C>T | |  |  |  |  | (Ostergaard et al., 2011) | | |
|  | Structural | NDUFS1 | c.1564C>A, c.1222C>T | |  |  |  |  | (Bugiani et al., 2004;Tuppen et al., 2010b) | | |
|  | Structural | NDUFS2 | c.671C>T, c.875T>C, c.866+4A>G | |  |  |  |  | (Bugiani et al., 2004) | | |
|  | Structural | NDUFS3 | c.418C>T, c.595C>T | |  |  |  |  | (Jaokar et al., 2013;Lou et al., 2018) | | |
|  | Structural | NDUFB8 | c.227C>A, c.432C>G | |  |  |  |  | (Piekutowska-Abramczuk et al., 2018a) | | |
|  | Structural | NDUFS4 | c.289or290G, c.316C>T | |  |  |  |  | (Budde et al., 2000) | | |
|  | Structural | NDUFS7 | c.434G>A | |  |  |  |  | (Bugiani et al., 2004;Lebon et al., 2007) | | |
|  | Structural | NDUFS8 | c.C236T, c.G305A | |  |  |  |  | (Loeffen et al., 1998) | | |
|  | Structural | NDUFV1 | c.1022C>T, c.1268C>T, c.640G>A, c.1294G>C, c.611A>G, c.616T>G | |  |  |  |  | (Schuelke et al., 1999;Zafeiriou et al., 2008;Vilain et al., 2012;Incecik et al., 2018) | | |
|  | Structural | NDUFV2 | c.IVS2 + 1delGTAA, c.669_670ins | |  |  |  |  | (Cameron et al., 2015) | | |
|  | Assembly | NDUFAF2 | c.114C>G | |  |  |  |  | (Hoefs et al., 2009) | | |
|  | Assembly | NDUFAF4 | c.194T>C | |  |  |  |  | (Saada et al., 2008;Baertling et al., 2017) | | |
|  | Assembly | NDUFAF5 (C20orf7) | c.477A>C | |  |  |  |  | (Gerards et al., 2010) | | |
|  | Assembly | NDUFAF6 | c.532G>C, c.420+784C>T, c.554_558del, c.371 T>C | |  |  |  |  | (Catania et al., 2018;Baide-Mairena et al., 2019) | | |
|  | Assembly | NDUFAF8 (C17ORF89) | c.45_52dup, c.1A>G, c.165C>G | |  |  |  |  | (Alston et al., 2020) | | |
|  | Assembly | FOXRED1 | c.1054C>T | |  |  |  |  | (Fassone et al., 2010;Zurita Rendon et al., 2016) | | |
|  | Assembly | NUBPL | c.166G>A, c.815-27T>C | |  |  |  |  | (Calvo et al., 2010) | | |

| **CII** | Structural | SDHA | c.1684C>T, c1660C>T, c.1A>C, c.1571C>T, c.248C>T, c.356G>A, c.1664G>A | CII deficiency, elevated lactate, succinate, pyruvate, SDH deficiency | Developmental delay with axial hypotonia, generalised muscular hypotonia with axial predominance, bilateral horizontal nystagmus, seizures, tetraparetic, rapidly progressive psychomotor regression, lack of speech development, spastic quadriparesis and partial loss of postural control with dystonia | (Bourgeron et al., 1995;Horvath et al., 2006;Pagnamenta et al., 2006) |
| --- | --- | --- | --- | --- | --- | --- |
|  | Assembly | SDHAF1 | c.169G>C, c.164G>C |  |  | (Ghezzi et al., 2009) |

| **CIII** | Structural | UQCRQ | c.208C>T | Mild to significantly elevated lactate levels, isolated CIII deficiency, increased citrate synthase, impaired respiration shown through decreased oxygen consumption | Severe psychomotor regression and extrapyramidal signs, dystonic postures, athetoid movements, ataxia, neurological regression, global dementia, progressive encephalopathy, early-onset developmental delay, spasticity, seizures, lactic acidosis, muscle hypotonia, failure to thrive, language regression, subacute rapid neurological failure | (Barel et al., 2008) |
| --- | --- | --- | --- | --- | --- | --- |
|  | Assembly | BCS1L | c.830G>A, c.296C>T, c.464, c.1,057G>A, c.217C>T, c.1102T>A, c.547C>T, c.550C>T |  |  | (de Lonlay et al., 2001;Fernandez-Vizarra et al., 2007;Baker et al., 2019) |
|  | Assembly | TTC19 | c.577G>A, c.964_967del |  |  | (Ghezzi et al., 2011;Atwal, 2014) |

| **C IV** | Structural | NDUFA4 | c.42+1G>C | Deficient COX activity in galactose but not glucose media, lactic acidosis, Isolated COX deficiency, citric acid cycle metabolites, glycine, and alanine, CIV deficiencies, elevated plasma alanine and proline, OXPHOS dysfunction associated with ATPsynthase defect and assembly | Bulbar dysfunction, dystonia, ataxia, spasticity, encephalopathy, pulmonary hypertension, recurrent vomiting, generalized epileptic tonic-clonic seizures, psychomotor retardation, microcephalus, enophthalmos, hypotonia, pigmentary retinopathy, developmental retardation and retrogression, hirsutism, growth retardation, nystagmus, hypoglycemia, metabolic acidosis, retinopathy, failure-to-thrive, hypertrophic cardiomyopathy, characteristic facial appearance, bilateral facial weakness, dysarthria and dysphagia | (Pitceathly et al., 2013) |
| --- | --- | --- | --- | --- | --- | --- |
|  | Structural | COX8A | c.115-1G>C |  |  | (Hallmann et al., 2016) |
|  | Assembly | SURF1 | c.743C>A, c.367_368del, c.772C>T, c.751C>T, c.833+1G>T, c.465_466del, c.532A>T, c.792_793del, c.845_846del, c.465_466del, c.826_827ins, c.532A>T |  |  | (Li et al., 2018) |
|  | Assembly | COX10 | c.791C>A, c.878C>T |  |  | (Antonicka et al., 2003) |
|  | Assembly | COX15 | c.C700T, c.503C>G, c.1081T>C |  |  | (Oquendo et al., 2004;Bugiani et al., 2005) |
|  | Assembly | SCO2 | c.418G>A |  |  | (Joost et al., 2010b) |
|  | Assembly | PET100 | c.3G>C |  |  | (Lim et al., 2014b) |
|  | Assembly | LRPPRC | c.1,119C>T |  |  | (Mootha et al., 2003;Debray et al., 2011;Mourier et al., 2014b) |
|  | Assembly | TACO1 | c.472C |  |  | (Weraarpachai et al., 2009b) |
| CV | Assembly | ATP5MD | c.87+1G>C | Elevated plasma alanine and lactate, reduction of CV dimerization, reduced ATP synthesis, | Gross motor developmental delay, gross motor regression, persistent hyperreflexia, bilateral plantar extensor signs, hypotonia, ocular movement abnormalities, ptosis, oro-motor incoordination, neurological regression, ataxia, hemiplegia, lethargic, bradycardia, | (Barca et al., 2018) |
