# Supplementary material for: Leigh Syndrome: A Tale of Two Genomes
Source: Front Physiol. 2021 Aug 11;12:693734. doi: 10.3389/fphys.2021.693734 (PMC8385445; doi:10.3389/fphys.2021.693734)
Supplement: Supplementary Table 2 — Mitochondrial genes involved in LS and LS-like disorders. [file Table_2.docx]

**Supplementary Table 2. Table showing mitochondrial genes involved in LS and LS-like disorders.** The most prevalent mutations affecting the different ETC complexes are summarized with the genetic defect and corresponding biochemical and clinical manifestations.

| **Mitochondrial genes involved in LS and LS-like disorders** | | | |  | |  |  | |  | |
| --- | --- | --- | --- | --- | --- | --- | --- | --- | --- | --- |
| **Complexes** |  | **Genes affected in LS** | **Genetic**  **defect** | **Biochemical**  **defect** | **Clinical manifestations** | | | **References** | |  |
| **CI** |  | MT-ND1 | m.3697G>A, m.3980G>A, m.3928G>C, m.3308T>C, m.3688G>A, m.3890G>A, m. 3460G>A, m.3946G>A | CI deficiency, elevated kreb cycle intermediate (especially fumarate and malate), elevated plasma and CSF lactate and pyruvic acid level, elevated alanine, Increased CI and CII activity, Elevated valine, isoleucine and lysine levels, elevated 3-ketoglutaric acid levels | Ataxia, developmental delay, lactic acidosis, psychomotor developmental retardation, cardiomyopathy, dystonia, respiratory failure, progressive encephalomyopathy, strabismus convergens, scoliosis, hypotonia, myoclonic jerks, pyramidal syndrome, bradypnoea, bradycardia, ptosis, ophthalmoplegia, proteinuria, haematuria, tubulointerstitial nephropathy, hypertrophic cardiomyopathy, peripheral neuropathy, optic atrophy, diabetes mellitus, nystagmus, epilepsy, stroke‐like episodes, cerebral paresis, facial weakness, impaired hearing, metabolic acidosis, drowsiness, vomiting, myoclonic jerks, hypothermia, stroke, oralmotor dyspraxia, spasticity, hemiparesis, dysarthria, drowsiness, vomiting, facial dyskinesia, generalized seizures, episodic central apneas | | | (Campos et al., 1997;Hinttala et al., 2006;Moslemi et al., 2008;Caporali et al., 2013;Wray et al., 2013;Negishi et al., 2014;Lee et al., 2016;Spangenberg et al., 2016;Ogawa et al., 2017) | |  |
|  |  | MT-ND2 | m.4681T>C, m.4833A>G |  |  |  |  | (Hinttala et al., 2006;Ugalde et al., 2007;Ma et al., 2013;Lee et al., 2016) | |  |
|  |  | MT-ND3 | m.10158T>C, m.10191T>C, m.10197G>A, m.10254G>A, 10134C>A |  |  |  |  | (Taylor et al., 2001b;Lebon et al., 2003;Bugiani et al., 2004;Crimi et al., 2004;McFarland et al., 2004b;Sarzi et al., 2007b;Lim et al., 2009;Naess et al., 2009;Leshinsky-Silver et al., 2010;Miller et al., 2014;Han et al., 2015;Lee et al., 2016;Li et al., 2019) | |  |
|  |  | MT-ND4 | m.11777C>A, m.11984T>C, m.11240C>T, m.11246G>A, m.11778G>A |  |  |  |  | (Komaki et al., 2003;Bugiani et al., 2004;Vanniarajan et al., 2006;Hadzsiev et al., 2010;Lee et al., 2016;Xu et al., 2017;Yu et al., 2018) | |  |
|  |  | MT-ND5 | m.12706T>C, m.13513G>A, m.13084 A>T, m.13511A>T, m.13042G>A, m.13094T>C, m.12338T>C, m.13514A>G |  |  |  |  | (Corona et al., 2001b;Taylor et al., 2002a;Crimi et al., 2003;Kirby et al., 2003b;Petruzzella et al., 2003;Blok et al., 2007;Ruiter et al., 2007;Shanske et al., 2008;Ching et al., 2013;Ma et al., 2013;Han et al., 2015;Lee et al., 2016) | |  |
|  |  | MT-ND6 | m.14459G>A, m.14487T>C, m.14439G>A, m.14502T>C |  |  |  |  | (Kirby et al., 2000b;Lebon et al., 2003;Bugiani et al., 2004;Gropman et al., 2004;Wang et al., 2009;Ronchi et al., 2011b;Tarnopolsky et al., 2013;Uehara et al., 2014) | |  |

| **CIII** |  | MT-CYB | m.14792C>G | Unlikely to have pathogenic significance | Unlikely to have pathogenic significance | (Andreu et al., 1999;Ronchi et al., 2011b) |
| --- | --- | --- | --- | --- | --- | --- |

| **CIV** |  | COXIII | m.9537Cinsertion, m.9952G>A | Normal CI & CII activity, elevated lactate, pyruvate, and alanine levels, decreased COX activity | Lactic acidosis, tetraparesis, ophthalmoparesis, convergent strabismus, reduced visual acuity and moderate mental retardation | (Keightley et al., 1996;Hanna et al., 1998;Tiranti et al., 2000) |
| --- | --- | --- | --- | --- | --- | --- |

| **CV** |  | MT-ATP6 | m.8993T>C/G, m.9176T>C/G, m.9185T>C | Normal enzyme activity in some patients, Abnormal/normal ATP synthesis, Abnormal MMP, Increased ROS, Abnormal sensitivity to oligomycin, Impaired CV assembly | Ataxia, bulbar palsy, pyramidal tract involvement, stroke-like episodes, seizures | (Santorelli et al., 1993a;Vilarinho et al., 2000;Carrozzo et al., 2001;Ogawa et al., 2017;Piekutowska-Abramczuk et al., 2018c;Wei et al., 2018;Ganetzky et al., 2019) |
| --- | --- | --- | --- | --- | --- | --- |
